# *Ciita* Regulates Local and Systemic Immune Responses in a Combined rAAV-*α*-synuclein and Preformed Fibril-Induced Rat Model for Parkinson’s Disease

**DOI:** 10.3233/JPD-240062

**Published:** 2024-06-04

**Authors:** Filip Fredlund, Itzia Jimenez-Ferrer, Kathleen Grabert, Lautaro Francisco Belfiori, Kelvin Luk, Maria Swanberg

**Affiliations:** a Department of Experimental Medical Science, Translational Neurogenetics Unit, Lund University, Lund, Sweden; b Department of Clinical Sciences, Inflammation and Stem Cell Therapy Group, Division of Clinical Neurophysiology, Lund University, Lund, Sweden; c Institute of Environmental Medicine, Toxicology Unit, Karolinska Institutet, Stockholm, Sweden; d Department of Pathology and Laboratory Medicine, Center for Neurodegenerative Disease Research, University of Pennsylvania Perelman School of Medicine, Philadelphia, PA, USA

**Keywords:** Parkinson’s disease, MHC class II transactivator protein, MHC class II genes, alpha-synuclein, tumor necrosis factor, neuroinflammation

## Abstract

**Background:**

Parkinson’s disease (PD) is characterized by alpha-synuclein (*α*-Syn) pathology, neurodegeneration and neuroinflammation. Human leukocyte antigen (*HLA*) variants associated with PD and *α*-Syn specific CD4+ T lymphocytes in PD patients highlight the importance of antigen presentation in PD etiology. The class II transactivator (CIITA) regulates major histocompatibility complex class II (MHCII) expression. Reduced *Ciita* levels significantly increase *α*-Syn pathology, nigrostriatal neurodegeneration and behavioral deficits in *α*-Syn-induced rat PD models.

**Objective:**

Characterize immune profiles associated with enhanced PD-like pathology observed in rats expressing lower *Ciita* levels (DA.VRA4) compared to the background strain (DA).

**Methods:**

To model PD, we combined rAAV-mediated *α*-Syn overexpression in the substantia nigra with striatal injection of *α*-Syn preformed fibrils. Immune profiles in brain and blood were analyzed by flow cytometry and multiplexed ELISA in naïve rats, 4- and 8 weeks post rAAV injection.

**Results:**

Flow cytometry showed *Ciita*-dependent regulation of MHCII on microglia, brain macrophages and circulating myeloid cells. The MHCII-dependent microglial response was highest at 4 weeks post rAAV injection, whereas the MHCII levels in circulating myeloid cells was highest at 8 weeks. There was no major infiltration of macrophages or T lymphocytes into the CNS in response to *α*-Syn and only subtle *Ciita*- and/or *α*-Syn-dependent changes in the T lymphocyte compartment. Lower *Ciita* levels were consistently associated with higher TNF levels in serum.

**Conclusions:**

*Ciita* regulates susceptibility to PD-like pathology through minor but detectable changes in resident and peripheral immune cells and TNF levels, indicating that mild immunomodulatory therapies could have therapeutic effects in PD.

## Plain Language Summary

Parkinson’s disease is characterized by loss of nerve cells. There is also abnormal aggregation of a protein called alpha-synuclein and an ongoing inflammatory response. Findings that immune cells in the blood of individuals with Parkinson’s disease react against the alpha-synuclein protein and that genes important for the immune system affect the risk of developing Parkinson’s disease indicate that immune responses are important in Parkinson’s disease. We have previously found that a low expression of certain immune molecules worsens disease progression in a rat model of Parkinson’s disease. The aim of this study was to identify changes in the immune system in rats that are associated with disease severity, to identify mechanisms that could be targeted to treat Parkinson’s disease. To model Parkinson’s disease, we injected a modified virus to produce large amounts of alpha-synuclein combined with an injection of aggregated alpha-synuclein proteins in the rat brain. The model mimics several features of Parkinson’s disease including nerve cell death, problems with movement, accumulation of alpha-synuclein in the brain, and an immune response. We observed that the immune system in the brain and blood responded to the model but that differences were small compared to controls. Our results suggest that small changes in the immune system can have a large effect on disease progression and that therapies targeting the immune system are worth exploring to find better treatment for Parkinson’s disease.

## INTRODUCTION

Parkinson’s disease (PD) is a progressive and incurable neurodegenerative disorder estimated to affect 2–3% of the population above the age of 65 [1]. Since the large majority of all PD cases have a multifactorial etiology, where genetics, lifestyle, and environment are contributing factors, the disease pathophysiology is complex [2]. A characteristic feature of PD is the degeneration of dopaminergic neurons in the substantia nigra pars compacta (SN), intraneuronal inclusions containing alpha-synuclein (*α*-Syn), and neuroinflammation [3]. The neuroinflammatory process includes microglial activation, local upregulation of major histocompatibility complex II (MHCII), altered levels of pro-inflammatory cytokines in cerebrospinal fluid (CSF) and blood, as well as systemic changes in lymphocyte populations [3]. Genetic association studies have identified single nucleotide polymorphisms in the human leukocyte antigen (*HLA*) locus that regulate the expression of MHCII to be associated with an increased risk of developing PD [4–6]. Recently, coding polymorphisms causing amino-acid changes in *HLA-D* haplotypes (HLA-DRB1*4) were also shown to be associated to PD with a protective effect [7]. Collectively, this indicates that both the quantity and quality of MHCII are involved in PD etiology. Since MHCII molecules present antigens to T lymphocytes and induce antigen-specific responses they serve as a link between the innate and adaptive immune systems [8].

A role of the adaptive immune system in PD etiology is supported by the presence of lymphocytes in postmortem brain tissue from PD patients [9] and findings of *α*-Syn reactive CD4+ lymphocytes [10, 11] early in the disease process [12]. However, it is not clear if and how antigen presentation contributes to or protects from PD pathology. The level of MHCII on antigen-presenting cells is controlled by the class II transactivator (CIITA, also known as MHC2TA) and *in vivo* silencing of *Ciita* using shRNA has been shown to prevent neurodegeneration in a nigral *α*-Syn overexpression model of PD in mice [13]. In contrast, we have previously found that congenic rats with lower *Ciita* and MHCII levels due to naturally occurring variants in the *Ciita* gene promotor have more widespread *α*-Syn pathology, more nigrostriatal neurodegeneration, more activated microglia and enhanced motor deficits after nigral overexpression of *α*-Syn alone [14] or combined with striatal seeding with *α*-Syn preformed fibrils (PFF) [15]. Of note, genetic variants mediating lower *Ciita* gene and MHC-protein expression are also found in humans and are associated with increased susceptibility to multiple sclerosis, rheumatoid arthritis and myocardial infarction, further adding to the interest of studying *Ciita* in relation to PD [16].

The aim of this study was to investigate the effect of *Ciita* expression on peripheral and local immune responses during *α*-Syn seeded PD-like pathology. To do so, we used a recombinant adeno-associated viral vector (rAAV) nigral *α*-Syn overexpression rat model combined with striatal seeding of human PFF in two rat strains with different susceptibility to PD-like pathology due to different transcriptional activity of the *Ciita* gene. The congenic DA.VRA4 strain has lower transcription of *Ciita* and *Mhc2*-genes and increased susceptibility to PD-like pathology compared to the background strain, DA. Using flow cytometry, we investigated both brain- and peripheral immune populations. We confirmed previous results that DA.VRA4 rats have lower MHCII expression in microglial cells compared to DA rats and we observe the highest microglia response at 4 weeks post rAAV injection in both strains. In addition to the local effects, we found lower MHCII levels on circulating myeloid cells, subtle changes in CD4+/CD8+ T-lymphocyte proportions in blood as well as higher levels of tumor necrosis factor (TNF) in serum in DA.VRA4 rats. Collectively, these results suggest that the levels of *Ciita* alter distinct immune populations and cytokine levels that in turn affect the susceptibility and severity of PD.

## MATERIALS AND METHODS

### Experimental design

**Fig. 1:**
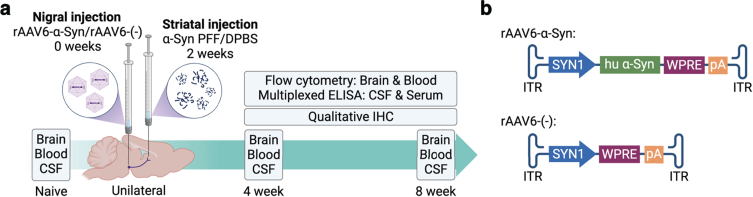
Experimental outline. **a.** Unilateral PD-like pathology was induced in DA and DA.VRA4 rats by rAAV6-mediated overexpression of human *α*-Syn in substantia nigra (week 0) combined with striatal seeding of human preformed fibrils of *α*-Syn (PFF, week 2). A control vector without the human *α*-Syn transgene combined with vehicle was used as control (rAAV6-(–)+DPBS). Brain, blood and CSF were collected from naïve rats, at 4- and 8-weeks post nigral injection. Immune populations in brain and blood were characterized by flow cytometry and cytokine levels in serum and CSF were analyzed by multiplexed ELISA. Qualitative immunohistochemistry was performed to assess *α*-Syn pathology, as well as the level of neurodegeneration and microglial activation. **b.** Schematic of the rAAV6-*α*-Syn vector used to overexpress human *α*-Syn in the substantia nigra and the control rAAV6-(–) vector without a transgene overexpression.

To investigate the effects of differential expression of *Ciita* we used wild type DA rats and a congenic DA.VRA4 rat strain with lower levels of *Ciita* and MHCII [14]. Male rats entered the study at 12±1 weeks of age and a total of 77 rats were included with 6–9 rats/group. We used a combination of viral overexpression of human *α*-Syn combined with seeding of human PFF, adapted from Thakur et al. [17]. Rats were injected with a rAAV6 vector carrying human *α*-Syn [18] into the SN followed two weeks later by an injection of human *α*-Syn PFF in the striatum (Fig. 1a) [15]. A rAAV6 vector without transgene and vehicle (Dulbecco’s phosphate buffered saline, DPBS) was used as control (Fig. 1b). Animals were sacrificed at 4- and 8-weeks post nigral injection for collection of brain, blood (whole blood and serum) and CSF samples. The 8-week timepoint was selected based on our previous work investigating the effects of differential *Ciita* expression on PD-like *α*-Syn pathology, neurodegeneration and neuroinflammation using the rAAV-*α*-Syn+PFF model [15]. Since there is an ongoing inflammatory response prior to the observed neurodegeneration in murine PD models [19–21] and in PD patients [11] we chose to include a 4-week timepoint as well. 6 animals per strain and timepoint were used to analyze brain and blood samples by flow cytometry and 2-3 animals per strain and timepoint were used for qualitative IHC validation of *α*-Syn expression, tyrosine hydroxylase (TH) loss, *α*-Syn pathology and MHCII upregulation. Naïve rats (*n* = 6 per strain) were sacrificed at 12±1 weeks of age. Two rats (one naïve DA and one DA.VRA4 *α*-Syn 8 week) were excluded from flow cytometry analysis of brain due to unsatisfactory perfusion and blood-filled ventricles, respectively. One DA rat from the 8-week *α*-Syn group was excluded from all analyses due to a clogged capillary during stereotactic surgery. One DA rat from the 8-week control group was excluded for flow cytometry analysis of blood due to inadequate number of events.

### Animals

The DA.VRA4 strain was generated by transfer of the VRA4 locus from the PVG strain to a DA background, resulting in two identical strains except for the VRA4 locus, the congenic strain (DA.VRA4) and the background strain (DA) [22]. DA.VRA4 and DA founders were provided by Professor Fredrik Piehl. Rats were housed 2-3 per cage in “type III high” individually ventilated cages with free access to standard rodent chow and water and kept in a pathogen-free and climate-controlled environment with a 12-h light/dark cycle at the Biomedical Center in Lund. All procedures were approved by the local ethics committee in the Malmö-Lund region and specified in permit 18037-19.

### Viral vectors

rAAV6 carrying human *α*-Syn under transcriptional regulation by the Synapsin-1 promotor and the woodchuck hepatitis virus posttranscriptional regulatory element [18] was generated as previously described [23] and injected at a concentration of 1.3E+10 gc/*μ*l (referred to as rAAV-*α*-Syn). The same vector but with the human *α*-Syn transgene excised was used as a control and injected at a concentration of 1.7E+10 gc/*μ*l (referred to as rAAV-(-)). The rAAV6 capsid concentrations were not quantified. The concentration of viral vectors was determined by ITR-qPCR.

### Preformed fibrils

Human *α*-Syn PFF were produced as previously described [24] and stored at –80°C until use. PFF were diluted to a concentration of 2.5*μ*g/*μ*l in sterile DPBS and sonicated for 6 min with 1 s ON/1 s OFF pulses at 70% power using a Q125 sonicator and cup horn (Qsonica, U.S.). The gross structure of PFF before and after sonication were imaged using transmission electron microscopy. PFF were diluted to a concentration of 0.025*μ*g/*μ*l and transferred to a hexagonal pattern 400 mesh cupper grid with a pioloform film reinforced with a carbon coat, for 20 min at room temperature (RT). Samples were stabilized with uranyl acetate for 1 min. Excess uranyl acetate was removed and the grids were left to dry for at least 5 min prior to imaging using a FEI Tecnai Spirit BioTWIN transmission electron microscope (FEI, U.S.).

### Surgical procedure

Rats were anaesthetized with 5% and maintained with 1–3% isoflurane (Isoflo vet, Orion Pharma) with a 2 : 1 mixture of O_2_: NO_2_ during the surgical procedure. Rats were attached to a stereotactic frame with a flat-skull position and 0.2 ml. Marcain (2.5 mg/ml, Aspen Nordic, Denmark) was subcutaneously (s.c.) injected under the scalp for local analgesia. Burr holes were created using a dental drill. For nigral injections, 3*μ*l rAAV6-(–) or rAAV6-*α*-Syn was injected in the following coordinates taken from bregma [25]; Anterior/posterior (A/P) – 5.3 mm, medial/lateral (M/L)±1.7 mm and dorsal/ventral (D/V) – 7.2 mm. For striatal injections, 3*μ*l of sonicated PFF (2.5*μ*g/*μ*l) or DPBS as control was injected using the following coordinates relative to bregma [25]; A/P – 0.4 mm, M/L±3.0 mm, and D/V – 4.5 mm. Injections were made unilaterally in the right hemisphere (ipsilateral) using a 10*μ*l Hamilton syringe (Hamilton, U.S.) fitted with a glass capillary. Injections were made with a flow rate of 0.5*μ*l/2 min and the capillary was left for 2 min after the injection before it was slowly retracted. The wound was sutured using surgical staples. Metacam (1 mg/kg) (Boehringer Ingelheim Animal Health, Germany) was injected s.c. for post-operative analgesia. The rats were left to recover in clean cages and monitored for 48 h post-surgery.

### Tissue collection and preparation

Rats were euthanized by intraperitoneal injection of 200–300 mg/kg sodium pentobarbital (APL, Sweden). Samples were collected in the following order: CSF, blood (serum and whole blood) and brain. CSF and blood were collected prior to perfusion whereas brain samples were collected after perfusion.

### CSF, serum, and whole blood collection and preparation

CSF samples were collected by attaching the rats in a stereotactic frame with an approximate 50–60° downward flex of the head. A midline incision was made over the neck and muscles covering the cisterna magna were severed using a scalpel. CSF samples were aspirated using a 27 G scalp vein set (Vygon, France) by inserting the bevel of the needle perpendicular to the cisterna magna. CSF was collected into protein LoBind tubes (Eppendorf, Germany), immediately put on dry ice and stored at –80°C until cytokine analysis. CSF samples contaminated with blood were excluded from analysis.

Blood was collected by cardiac puncture. Serum was prepared by leaving whole blood undisturbed at RT for 30–60 min followed by centrifugation for 10 min at 4°C and 2,000xg. Serum was aliquoted into protein LoBind tubes (Eppendorf, Germany) and stored at –80°C until cytokine analysis.

Whole blood was collected into K3E EDTA coated tubes (BD, U.S.) and stored at 4°C for 3–4 h until preparation for flow cytometry. Red blood cells were lysed by adding 1.8 ml of 1x Pharm Lyse (BD, U.S.) to 200*μ*l whole blood samples and incubated at RT for 15–20 min. Cells were washed in sterile-filtered PBS (pH 7.2) and resuspended in sterile-filtered ice-cold fluorescence-activated cell sorting (FACS) buffer (2% (w/v) bovine serum albumin fraction V (Roche, Switzerland) and 0.01% sodium azide (w/v) in PBS (pH 7.2)) before proceeding with antibody staining for flow cytometry.

### Brain collection and processing for immunohistochemistry and flow cytometry

After CSF and blood sampling, rats were transcardially perfused with 0.9% saline (w/v) with the descending aorta clamped using hemostatic forceps for at least 5 min or until no blood was visible. For IHC analysis, rats were subsequently perfused with ice-cold 4% paraformaldehyde (PFA) (w/v) for 5 min and the brains post-fixed in 4% PFA (w/v) at 4°C overnight followed by cryopreservation in PBS containing 30% sucrose (w/v) and 0.01% sodium azide (w/v), pH 7.2 until sectioning. For flow cytometry experiments, saline-perfused brains were collected into ice-cold Roswell Park Memorial Institute 1640 medium without phenol red (Gibco/Thermo Fischer Scientific, U.S.) and stored at 4°C for a maximum of 3 h until processing.

For flow cytometry experiments, hemispheres of freshly collected brains were separated and put into a 7 ml glass Dounce tissue grinder (DWK, Germany) with 3–5 ml ice-cold 1x Hank’s Balanced Salt Solution without calcium, magnesium or phenol red (HBSS) (Gibco/Thermo Fischer Scientific, U.S.), pH 7.0–7.4. Each hemisphere was homogenized on ice using the large clearance pestle followed by the small clearance pestle until complete homogenization. The glass Dounce tissue grinder set was washed with detergent, rinsed and dried between samples. Homogenized samples were passed through a 100*μ*m nylon cell strainer (Falcon, U.S.) into a 50 ml conical tube to remove any remaining large debris. 1x HBSS (pH 7.0–7.4) was added until a total volume of 12 ml was reached and samples were kept on ice until separation of myelin and brain mononuclear cells.

Brain mononuclear cells were isolated and myelin removed using an adapted two-layer density gradient protocol [26, 27]. A 100% stock isotonic Percoll (SIP) was prepared by diluting Percoll (GE Healthcare, U.S.) 9 : 1 in 10x HBSS (Gibco/Thermo Fischer Scientific, U.S.) and 35% SIP was prepared by diluting 100% SIP 0.35 : 1 in 1x HBSS pH 7.0–7.4. Homogenized brain samples were centrifuged for 5 min at 4°C and 400xg, the supernatant was discarded and the pellet was thoroughly resuspended in 16 ml of 35% SIP. The cell suspension was carefully layered with 5 ml of 1x HBSS pH 7.0–7.4 and centrifuged for 30 min at 4°C and 800xg without brake. The HBSS layer (top), myelin layer (between HBSS and 35% SIP) and 35% SIP was aspirated and the pelleted isolated brain mononuclear cells were washed in 10 ml of 1x HBSS pH 7.0–7.4 and resuspended in ice-cold FACS buffer.

### Antibody staining for flow cytometry

Fc*γ*II receptors on blood and brain samples were blocked by adding anti-rat CD32 diluted 1 : 200 and incubated for 5 min at 4°C. 50*μ*l of cell suspension was stained using an antibody cocktail (Table 1) diluted in Brilliant Stain Buffer (BD, U.S.). Cells were incubated with antibodies for 30 min at 4°C in dark followed by washing in sterile PBS (pH 7.2). Cells were resuspended in 250*μ*l of sterile FACS buffer containing DRAQ7 diluted 1 : 1,000 prior to analysis.

**Table 1:** Antibodies, viability marker and compensation beads used for flow cytometry

Antigen/Target	Species specificity	Fluorochrome/Conjugation	Clone	Isotype/Host	Dilution	Company
CD45	Rat	APC-eFluor 780	OX1	Mouse IgG1, *κ*	1 : 100	Invitrogen (47-0461-82)
CD3	Rat	BV421	1F4	Mouse IgM, *κ*	1 : 200	BD Horizon (563948)
CD4	Rat	BV605	OX-35	Mouse IgG2a, *κ*	1 : 200	BD OptiBuild (740369)
CD8a	Rat	PE-Cy7	OX8	Mouse IgG1, *κ*	1 : 200	Invitrogen (25-0084-82)
CD11b	Rat	PE	WT.5	Mouse IgA, *κ*	1 : 200	BD Pharmingen (562105)
MHCII RT1B	Rat	Alexa Fluor 647	OX-6	Mouse IgG1, *κ*	1 : 400	Bio-Rad (MCA46A647)
CD86	Rat	BV711	24F	Mouse IgG1, *κ*	1 : 100	BD OptiBuild (743215)
Fc*γ*RII	Rat	–	D34-485	Mouse IgG1, *κ*	1 : 200	BD Pharmingen (550270)
Compensation	Mouse, *κ*	–	–	–	–	BD CompBeads (552843)
Viability/dsDNA	–	DRAQ7	–	–	1 : 1,000	Invitrogen (D15106)

Samples were analyzed using an LSR Fortessa (BD, U.S.), configuration specified in Table 2. Compensation was performed using BD CompBeads (BD, U.S.) and prepared according to manufacturer’s instructions. Fluorescence minus one, unstained cells and unstained cells with viability dye were included for each recording session and for each sample type (blood or brain) and used to set gates. Gating strategy for brain and blood samples can be seen in Supplementary Figures 2a and 3a. Microglial cells were gated as CD45^dim^CD11b+ in brain samples. Infiltrating macrophages/monocytes (CD45^high^CD11b+) and T lymphocytes (CD45+CD3+) in brain samples were rare with <1,000 events/hemisphere. Myeloid population in blood was gated as CD45+CD11b+ and T lymphocytes as CD45+CD3+. T helper cells were gated as CD4+ and cytotoxic T lymphocytes as CD8+. Data was analyzed using FlowJo software version 10.8.1 (BD, U.S.). All analyses were done on freshly isolated tissue and recorded during multiple sessions. 4–6 rats were used at each recording session (equal number of DA and DA.VRA4 rats per session) from the same experimental group (naïve/control/*α*-Syn) and timepoint (4- or 8- weeks).

**Table 2:** Configuration of the LSR Fortessa and filters used for recording of isolated blood and brain cells by flow cytometry

Laser	Filter	Fluorochrome
Blue – 488 nm	780/60	PE-Cy7
	695/40	–
	610/20	–
	575/26	PE
	530/30	–
	488/10	SSC
Red – 640 nm	780/60	APC-eFluor 780
	730/45	DRAQ7
	670/30	Alexa Fluor 647
Violet – 405 nm	780/60	–
	710/50	BV711
	660/20	–
	610/20	BV605
	525/50	–
	442/46	BV421

### Immunohistochemistry

Fixed brains were coronally sectioned on a Microm HM450 freezing microtome (Thermo Scientific, U.S.) with 35*μ*m thickness in series of 12 and stored in Walter’s antifreeze solution at 4°C until IHC staining. All stainings were done on free floating sections except for proteinase K treated human *α*-Syn staining which was done on sections mounted on gelatin-coated glass slides. Sections were rinsed with PBS or 0.1% PBS with Triton-X 100 (v/v) (PBST) between all incubation steps. For proteinase K resistant *α*-Syn aggregates, sections were incubated with 5*μ*g/ml Proteinase K (Thermo Fischer Scientific, U.S.) diluted in TBS for 1 h at RT prior to quenching. For 3,3'-diaminobenzidine (DAB) staining sections were quenched with 3% H_2_O_2_ (v/v) and 10% MetOH (v/v) in PBS. Sections were blocked with 10% serum (same species as secondary antibody) in 0.3% PBST. Primary antibody was diluted in 0.3% PBST with 5% serum (same species as secondary antibody) and incubated at 4°C overnight. On the following day sections were incubated with biotinylated secondary antibody and incubated for 1 or 2 h at RT (DAB or Fluorescence, respectively). All antibodies used for IHC are found in Table 3. For DAB staining, horseradish peroxidase conjugated avidin/biotin-complex (Vector laboratories, U.S.) was prepared according to manufacturer’s instructions and added to the sections for 30 min at RT. A DAB substrate kit (Vector laboratories, U.S.) was prepared according to manufacturer’s instructions and used as a chromogen for visualization. DAB sections were mounted on gelatin-coated glass slides, dehydrated and coverslipped using Pertex (Histolab, Sweden). Fluorescently stained sections were coverslipped using PVA/DABCO and stored at 4°C in dark. Brightfield overview images of TH and human *α*-Syn were acquired using an Olympus VS-120 virtual slide scanner (Olympus, Japan). Brightfield images of pS129 *α*-Syn and proteinase K treated human *α*-Syn in SN was acquired using an Olympus BX53 (Olympus, Japan). MHCII+ microglia cells were imaged using a Leica SP8 scanning confocal microscope (Leica, Germany).

**Table 3:** List of antibodies used for immunohistochemistry

Antigen/Secondary antibody	Host	Dilution	Company
Human *α*-Syn	Mouse	1 : 1,000	Santa Cruz (sc-12767)
Biotinylated anti-mouse	Horse	1 : 200	Vector Laboratories (BA-2001)
TH	Rabbit	1 : 1,000	EMD Millipore (AB152)
pS129 *α*-Syn	Rabbit	1 : 2,000	Abcam (ab51253)
Biotinylated anti-rabbit	Goat	1 : 200	Vector Laboratories (BA-1000)
MHCII	Mouse	1 : 500	Abcam (ab23990)
Alexa Fluor 488 anti-mouse	Donkey	1 : 200	Abcam (ab150105)

### Cytokine analysis

Cytokine analysis in serum and CSF was performed using the V-PLEX Proinflammatory panel 2 Rat Kit from Mesoscale diagnostics (MSD, U.S.) according to manufacturer’s instructions. The plates were washed using PBS with 0.05% Tween-20 between incubation steps. Serum samples were diluted 4-fold and CSF samples 2-fold, according to manufacturer’s recommendation. Plates were read on a MESO QuickPlex SQ 120 analyzer (MSD, U.S.). Results were analyzed using the Discovery Workbench software version 4.0.13 (MSD, U.S.). The number of samples used for cytokine analysis differs as a consequence of available wells on the MSD plate. All samples were run in duplicates and the mean value was used for analysis. If only one replicate was detected it was included in the analysis. If both replicates were below the lower limit of detection (LLOD) for a sample the non-detected (ND) value was replaced with the lowest quantifiable value for the specific cytokine. If duplicates for more than one sample was below the LLOD for a group, no statistical comparisons were made and presented as non-detected (ND).

### Statistical analyses

Statistical analyses were conducted using the GraphPad Prism software version 10.1.1 (San Diego, CA, U.S.). Quantile-quantile plot of residuals was used to determine the use of parametric or non-parametric tests. Data in figures is presented as mean±SD and individual values. Naïve rats were compared by unpaired Student’s t-test. Groups at 4- and 8-weeks were compared with two-way ANOVA with Šídák multiple comparison test (DA vs. DA.VRA4 and control vs. *α*-Syn). Data in text is presented as (mean1±SD1 vs. mean2±SD2, p-value, 95% CI of difference [lower limit, upper limit]). A significance level of *α*<0.05 was used for all analyses.

## RESULTS

### Qualitative immunohistochemistry confirms PD-like features characterized by *α*-Syn pathology and nigrostriatal dopaminergic neurodegeneration in the rAAV-*α*-Syn+PFF model

To investigate the effects of differential levels of *Ciita* on PD like-pathology we used the congenic DA.VRA4 rat strain with lower levels of *Ciita* and reduced expression MHCII [14] with DA rats as controls. Rats were injected with rAAV-*α*-Syn into the SN followed by an injection of sonicated human *α*-Syn PFF two weeks later in the striatum (*α*-Syn group) (Fig. 1 and Supplementary Figure 1a). Control animals were injected with rAAV-(–) into the SN and vehicle (DPBS) into the striatum (control group). Rats were sacrificed at baseline (naïve), 4- and 8-weeks post nigral injection for collection of brain, blood and CSF samples (Fig. 1a).

The rAAV-*α*-Syn+PFF model used has been thoroughly characterized in a previous study, showing significantly reduced striatal TH+ fiber density and motor deficits in DA.VRA4 but not DA rats, as well as more aggregation and spread of *α*-Syn in DA.VRA4 compared to DA rats [15]. Quantification of the same parameters are not ethically warranted and not within the scope of the current study. Therefore, qualitative histological assessment was done to confirm the model. Robust staining for of human *α*-Syn was observed at 4- and 8-weeks in *α*-Syn (Supplementary Figure 1b, c) but not in control groups (Supplementary Figure 1d, e). As expected, the unilateral rAAV-*α*-Syn+PFF model induced loss of TH+ signal in the ipsilateral SN and striatum of both DA and DA.VRA4 rats at 4- and 8-weeks (Supplementary Figure 1f, g), whereas the TH+ signal remained intact in the control groups (Supplementary Figure 1h, i). rAAV-*α*-Syn+PFF injection also resulted in pathological forms of *α*-Syn aggregates, represented by positive signal for *α*-Syn phosphorylated at serine residue 129 (pS129) in cell somas and neurites, as well as by proteinase K-resistant *α*-Syn aggregates mainly observed as puncta along neurites in ipsilateral, but not contralateral SN (Supplementary Figure 1 j, k). Additionally, rAAV-*α*-Syn+PFF injection lead to upregulation of MHCII molecules in the ipsilateral but not contralateral midbrain of both DA and DA.VRA4 rats (Supplementary Figure 1l).

### MHCII+ microglia response is highest at 4 weeks after rAAV-*α*-Syn+PFF and MHCII expression on microglia and brain macrophages is regulated by *α*-Syn and *Ciita*

**Fig. 2:**
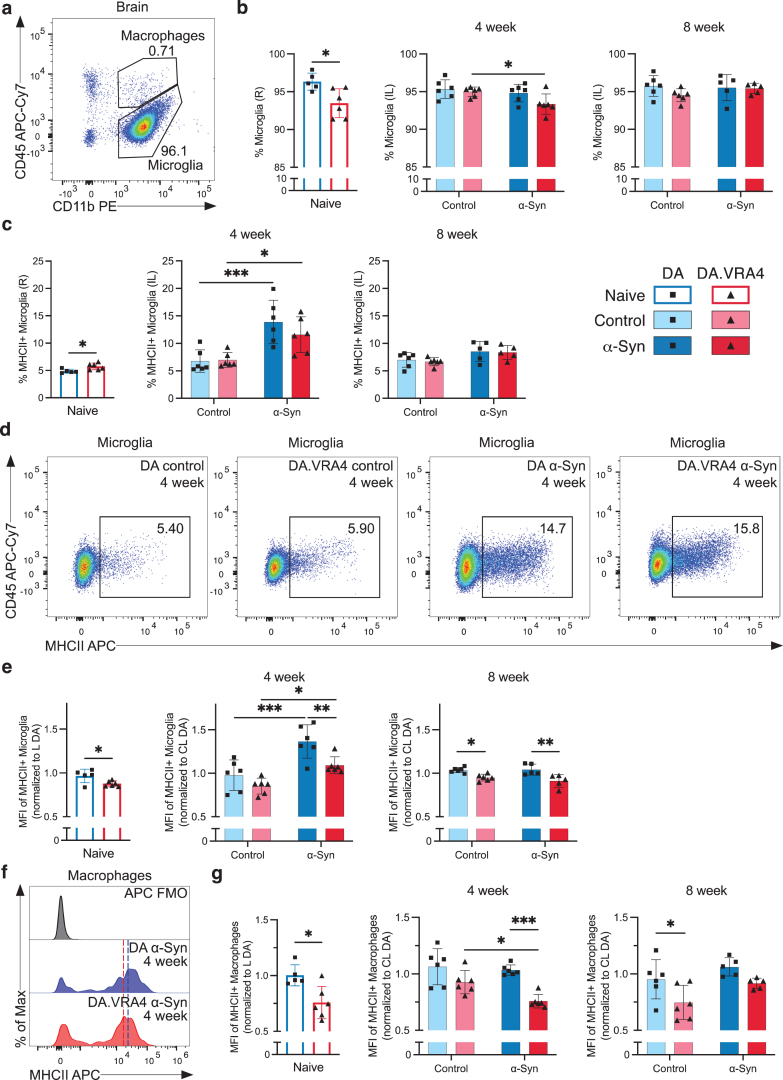
Effects of rAAV-*α*-Syn+PFF on microglia, brain infiltrating macrophages and MHCII expression. **a.** Gating of microglia (CD45^dim^CD11b+) and infiltrating macrophages/monocytes (CD45^high^CD11b+) in brain hemispheres. **b.** Quantification of microglia in right (R)/ipsilateral (IL) hemispheres. **c.** Quantification of MHCII+ microglia in R/IL hemispheres. **d.** Representative flow cytometry dot plots of MHCII+ microglia in IL hemispheres at the 4-week timepoint. **e.** Quantification of relative median fluorescence intensity (MFI) of MHCII+ microglia in R/IL hemispheres. At each recording session, R/IL MFI-values were normalized to the mean MFI-values in left (L)/contralateral (CL) hemisphere of DA. **f.** Representative histograms of MHCII expression on brain infiltrating macrophages with fluorescence minus one (FMO) control. The approximate MHCII+ MFI-value is indicated by dashed line; blue (DA) and red (DA.VRA4). **g.** Quantification of relative MFI of MHCII+ macrophages in R/IL hemispheres. As for microglia, R/IL MFI-values were normalized to the mean MFI-values of MHCII+ macrophages in L/CL hemisphere of DA for each recording session. **b**, **c**, **e** and **g**. Naïve (DA *n* = 5, DA.VRA4 *n* = 6), 4-week; control (DA *n* = 6, DA.VRA4 *n* = 6) and *α*-Syn (DA *n* = 6, DA.VRA4 *n* = 6), 8-week; control (DA *n* = 6, DA.VRA4 *n* = 6) and *α*-Syn (DA *n* = 5, DA.VRA4 *n* = 5). *α*-Syn=rAAV-*α*-Syn+PFF. Control = rAAV-(–)+DPBS. Naïve rats were compared by unpaired Student’s t-test. Groups at 4- and 8-weeks were compared with two-way ANOVA with $\overset{ˇ}{S}$ ídák multiple comparison test (DA vs. DA.VRA4 and control vs. *α*-Syn). ^*^p < 0.05, ^**^p < 0.01 and ^***^p < 0.001. Data presented as mean±SD with individual values.

Since DA.VRA4 rats with lower *Ciita* levels are more susceptible to *α*-Syn pathology and dopaminergic neurodegeneration than DA rats [14, 15], we characterized microglia, infiltrating macrophages and T lymphocyte populations in brain tissue by flow cytometry (Supplementary Figure 2a). DA.VRA4 and DA did not differ in terms of microglial population size in the ipsilateral hemisphere except for naïve DA.VRA4 rats which had a lower percentage of microglia compared to DA in the right hemisphere (93±1.9% vs. 96±1.1%, *p* = 0.017, 95% CI [–5.1, –0.65]) (Fig. 2a, b); however, this difference was not observed in the left hemisphere (data not shown). In DA.VRA4, a lower amount of microglia was observed in the *α*-Syn group compared to controls at 4 weeks (93±1.4% vs. 95±0.6%, *p* = 0.042, 95% CI [–3.2, –0.06]) (Fig. 2b).

In naïve DA.VRA4 rats, a larger proportion of microglia was MHCII+ compared to DA (5.7±0.60% vs. 4.9±0.35%, *p* = 0.019, 95% CI [0.18, 1.6]) (Fig. 2c). Although immunohistochemical staining has previously found increased numbers of activated microglia in the striatum of DA.VRA4 rats compared to DA [14, 15] the percentage of MHCII+ microglia in entire ipsilateral hemispheres did not differ between the strains at either 4-or 8-weeks (Fig. 2c). Compared to controls, rAAV-*α*-Syn+PFF injection lead to an expansion of MHCII+ microglia in both DA (14±4.0% vs. 6.8±2.0%, *p* < 0.001, 95% CI [3.2, 11]) and DA.VRA4 (12±3.2% vs. 7.0±1.3%, *p* = 0.021, 95% CI [0.65, 8.5]) at 4 weeks (Fig. 2c, d). By 8 weeks, the amount of MHCII+ microglia were reduced to baseline in both strains (Fig. 2c).

Quantification of MHCII on microglia (relative MFI-values; normalized to mean MHCII+ MFI in left/contralateral hemispheres of DA at each recording session) revealed that congenic DA.VRA4 rats had lower MHCII expression compared to DA in naïve rats (0.88±0.032 vs. 0.97±0.077, *p* = 0.029, 95% CI [–0.17, –0.011]), at 4 weeks in the *α*-Syn group (1.1±0.097 vs. 1.4±0.19, *p* = 0.008, 95% CI [–0.48, –0.070]) and at 8 weeks in both the control- (0.95±0.038 vs. 1.0±0.030, *p* = 0.019, 95% CI [–0.16, –0.014]) and *α*-Syn group (0.91±0.072 vs. 1.0±0.063, *p* = 0.002, 95% CI [–0.21, –0.05]) (Fig. 2e). Both strains had higher MHCII expression at 4 weeks in the *α*-Syn group compared to control (DA: 1.4±0.19 vs. 0.98±0.18, *p* < 0.001, 95% CI [0.19, 0.60] and DA.VRA4 : 1.1±0.10 vs. 0.85±0.09, *p* = 0.020, 95% CI [0.04, 0.44]) (Fig. 2e). The increase in MHCII levels observed in *α*-Syn group at 4 weeks returned to control levels again at the 8-week timepoint in both strains (Fig. 2e).

We did not observe any strain- or *α*-Syn-dependent changes in infiltrating macrophages/monocytes (CD45^high^CD11b+) populations in brain in terms of overall amount or percentage of MHCII+ macrophages (Supplementary Figure 2b, c). However, infiltrating macrophages in DA.VRA4 rats had lower expression (relative MFI levels) of MHCII+ compared to DA for naïve (0.76±0.15 vs. 1.0±0.094, *p* = 0.010, 95% CI [–0.42, –0.073]), *α*-Syn at 4 weeks (0.76±0.061 vs. 1.0±0.046, *p* < 0.001, 95% CI [–0.42, –0.13]) and control at 8 weeks (0.75±0.15 vs. 0.95±0.17, *p* = 0.026, 95% CI [–0.39, –0.024]) (Fig. 2f, g).

The expression (relative MFI levels) of CD86 (also known as B7-2, a co-stimulatory signal expressed by antigen-presenting cells necessary for activation of T lymphocytes [8]) on microglia and macrophages in the right/ipsilateral hemisphere did not differ between strains (Supplementary Figure 2d, e). There were, however, lower CD86 levels in DA *α*-Syn vs. control at 4 weeks (0.92±0.12 vs. 1.2±0.08, *p* = 0.004, 95% CI [–0.41, –0.08]) (Supplementary Figure 2e). No *α*-Syn- or strain-dependent differences were observed in percentages of infiltrating T lymphocytes in right/ipsilateral hemispheres (Supplementary Figure 2a, f).

### MHCII+ expression on blood myeloid cells and amount of CD3+ lymphocytes are regulated by *α*-Syn and *Ciita*

**Fig. 3:**
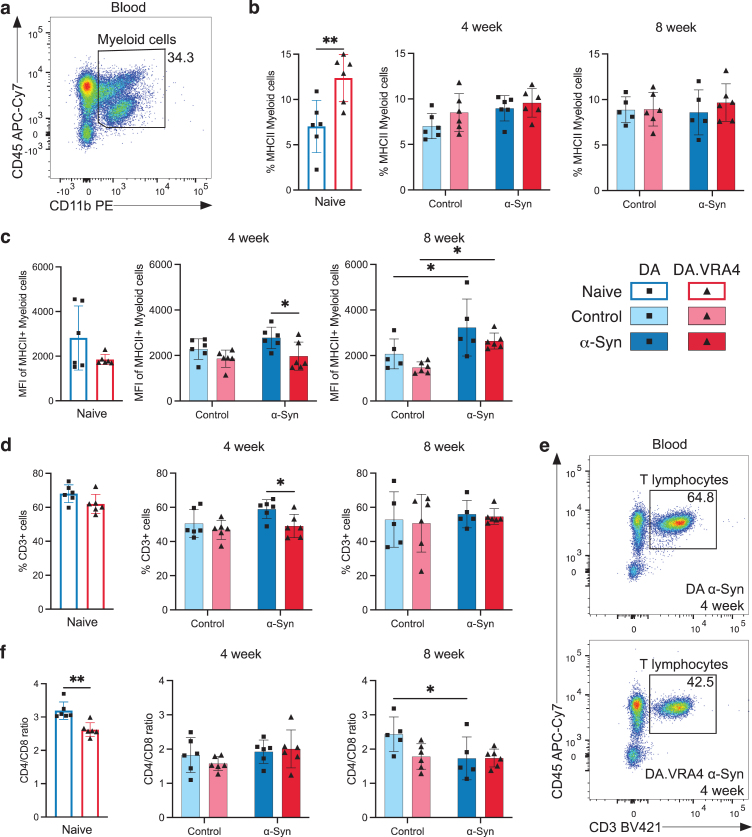
Systemic effects of rAAV-*α*-Syn+PFF on blood myeloid cells’ MHCII expression and circulating lymphocyte profiles. **a.** Gating of myeloid cells (CD45+CD11b+) in blood. **b.** Quantification of MHCII+ myeloid cells in blood. **c.** Quantification of MFI-values of MHCII+ myeloid cells in blood. **d.** Quantification of T lymphocytes (CD3+CD45+) in blood. **e.** Representative flow cytometry dot plot of circulating T lymphocyte population in DA (top) and DA.VRA4 (bottom) at 4 weeks in the *α*-Syn group. **f.** Quantification of CD4/CD8 ratio in blood. **b–d** and **f.** Naïve (DA *n* = 6, DA.VRA4 *n* = 6), 4 week; control (DA *n* = 6, DA.VRA4 *n* = 6) and *α*-Syn (DA *n* = 6, DA.VRA4 *n* = 6), 8 week; control (DA *n* = 5, DA.VRA4 *n* = 6) and *α*-Syn (DA *n* = 5, DA.VRA4 *n* = 6). *α*-Syn = rAAV-*α*-Syn+PFF. Control = rAAV-(–)+DPBS. Naïve rats were compared by unpaired Student’s t-test. Groups at 4- and 8-weeks were compared with two-way ANOVA with $\overset{ˇ}{S}$ ídák multiple comparison test (DA vs. DA.VRA4 and control vs. *α*-Syn). ^*^p < 0.05 and ^**^p < 0.01. Data presented as mean±SD with individual values.

To investigate changes in peripheral immune cell populations, we performed flow cytometry of blood collected 4- and 8-weeks post nigral injection (Supplementary Figure 3a). No strain- or *α*-Syn-dependent changes in overall percentage of myeloid (CD45+CD11b+) cells (Fig. 3a and Supplementary Figure 3b) or MHCII+ myeloid cells were observed, except from more MHCII+ myeloid cells in naïve DA.VRA4 compared to naïve DA (12±2.6% vs. 7.0±2.9%, *p* = 0.0071, 95% CI [1.8, 8.9]) (Fig. 3b). Similar to MHCII+ macrophages in the brain, blood macrophages in DA.VRA4 rats had lower intensity of MHCII signal compared to DA *α*-Syn group at 4 weeks (1970±620 vs. 2780±470 MFI, *p* = 0.019, 95% CI [–1510, –100]) (Fig. 3c). Unlike the early response in microglia, blood myeloid cells from both strains displayed a delayed MHCII+ response to *α*-Syn at 8 weeks in both strains (DA: 3230±1250 vs. 2070±660 MFI, *p* = 0.035, 95% CI [80, 2250] and DA.VRA4 : 2640±350 vs. 1480±250 MFI, *p* = 0.021, 95% CI [170, 2150]) (Fig. 3c). We found no *Ciita*- or *α*-Syn-dependent regulation of CD86 expression on blood myeloid cells (Supplementary Figure 3c).

The overall percentage of circulating T lymphocytes was lower in DA.VRA4 compared to DA rats in the *α*-Syn group at 4 weeks (49±6.8% vs. 59±5.6%, *p* = 0.021, 95% CI [–18, –1.9]) but did not differ at 8 weeks or between *α*-Syn and control groups (Fig. 3d, e). Among CD3+ lymphocytes, the CD4/CD8 ratio was lower in naïve DA.VRA4 compared to DA (2.6±0.21 vs. 3.2±0.26, *p* = 0.0019, 95% CI [–0.87, –0.27]) (Fig. 3f) which was driven by a decrease in CD4+ T lymphocytes (70±2.5% vs. 74±1.4%, *p* = 0.0037, 95% CI [–7.0, –1.8]) (Supplementary Figure 3d) and an increase in CD8+ T lymphocytes (27±1.5% vs. 23±1.4%, *p* = 0.0023, 95% CI [1.5, 5.2]) (Supplementary Figure 3e). Additionally, the CD4/CD8 ratio was reduced in response to *α*-Syn compared to control in DA rats at 8 weeks (1.7±0.63 vs. 2.4±0.50, *p* = 0.045, 95% CI [–1.4, –0.01]) (Fig. 3f), driven by an increase of CD8+ T lymphocytes (37±7.3% vs. 28±4.9%, *p* = 0.047, 95% CI [0.16, 18]) (Supplementary Figure 3e).

### Few changes in CSF and serum cytokines but consistently higher levels of serum TNF in DA.VRA4 rats with lower levels of *Ciita* compared to DA

Altered levels of cytokines in the CSF and serum have been reported in PD patients [3]. To investigate the effect of *Ciita* expression and rAAV-*α*-Syn+PFF injection on CSF and serum cytokine levels, we performed multiplexed ELISA. Most of the cytokines investigated were detectable in both CSF and serum samples, however, the cytokine levels were often below the lower limit of quantification, ultimately affecting the accuracy of the results (results summarized in Supplementary Tables 1–6).

**Fig. 4:**
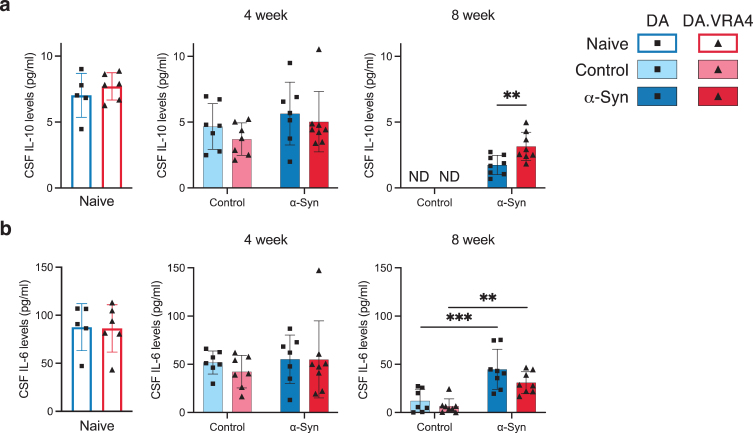
CSF IL-10 and IL-6 cytokine levels are regulated by differing *Ciita* levels and in response to rAAV-*α*-Syn+PFF, respectively. **a.** Quantification of IL-10 levels in cerebrospinal fluid (CSF) measured by ELISA. Non-detected levels indicated by “ND”. **b.** Quantification of IL-6 levels in CSF measured by ELISA. **a–b.** Naïve (DA *n* = 5, DA.VRA4 *n* = 6), 4-week; control (DA *n* = 7, DA.VRA4 *n* = 7) and *α*-Syn (DA *n* = 7, DA.VRA4 *n* = 8), 8-week; control (DA *n* = 7, DA.VRA4 *n* = 8) and *α*-Syn (DA *n* = 8, DA.VRA4 *n* = 8). *α*-Syn = rAAV-*α*-Syn+PFF. Control = rAAV-(–)+DPBS. Naïve rats were compared by unpaired Student’s t-test. Groups at 4- and 8-weeks were compared with two-way ANOVA with $\overset{ˇ}{S}$ ídák multiple comparison test (DA vs. DA.VRA4 and control vs. *α*-Syn). ^**^p < 0.01 and ^***^p < 0.001. Data presented as mean±SD with individual values.

Most cytokines analyzed in CSF levels were unaffected by *Ciita* and *α*-Syn, but we found differences in IL-10 and IL-6. In the *α*-Syn group at 8 weeks, DA.VRA4 rats had higher IL-10 CSF levels compared to DA (3.2±1.1 pg/ml vs. 1.7±0.74 pg/ml, *p* = 0.0084, 95% CI [0.42, 2.4]) (Fig. 4a). rAAV-*α*-Syn+PFF induced increased CSF levels of IL-6 at 8 weeks in both DA (45±21 pg/ml vs. 12±12 pg/ml, *p* < 0.001, 95% CI [16, 50]) and DA.VAR4 (31±11 pg/ml vs. 6.6±7.6 pg/ml, *p* = 0.003, 95% CI [8.1, 41]) (Fig. 4b).

**Fig. 5:**
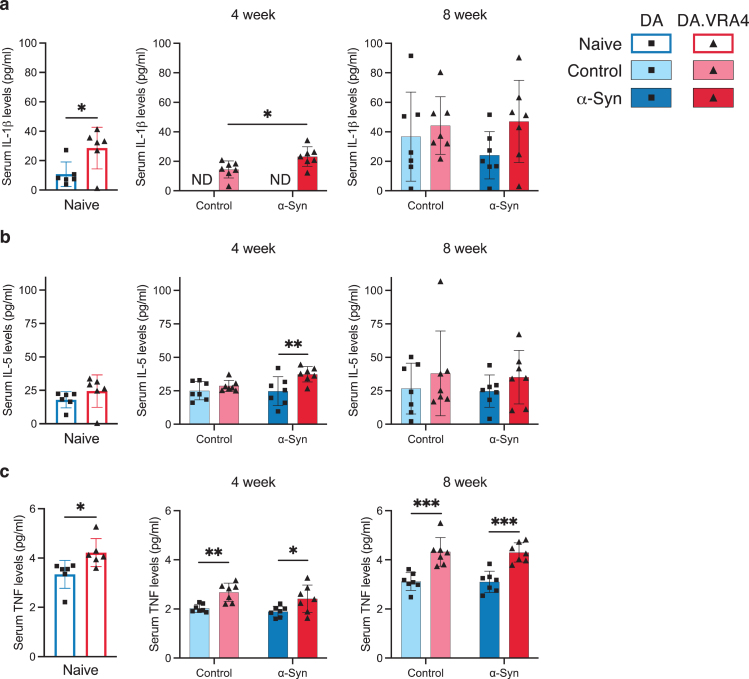
*Ciita* regulates TNF levels in serum. **a.** Quantification of serum IL-1β levels measured by ELISA. Non-detected levels indicated by “ND”. **b.** Quantification of serum IL-5 levels measured by ELISA. **c.** Quantification of TNF levels in serum measured by ELISA. **a–c.** Naïve (DA *n* = 6, DA.VRA4 *n* = 6), 4-week; control (DA *n* = 7, DA.VRA4 *n* = 7) and *α*-Syn (DA *n* = 7, DA.VRA4 *n* = 7), 8-week; control (DA *n* = 7, DA.VRA4 *n* = 7) and *α*-Syn (DA *n* = 7, DA.VRA4 *n* = 7). *α*-Syn = rAAV-*α*-Syn+PFF. Control = rAAV-(–)+DPBS. Naïve rats were compared by unpaired Student’s t-test. Groups at 4- and 8-weeks were compared with two-way ANOVA with $\overset{ˇ}{S}$ ídák multiple comparison test (DA vs. DA.VRA4 and control vs. *α*-Syn). ^*^p < 0.05, ^**^p < 0.01 and ^***^p < 0.001. Data presented as mean±SD with individual values.

In serum, we found differences in IL-1β, IL-5, and TNF levels. IL-1β levels were higher in naïve DA.VRA4 compared to DA (29±14 pg/ml vs. 11±8.3 pg/ml, *p* = 0.025, 95% CI: [2.8, 33]) and at 4 weeks in the *α*-Syn group in DA.VRA4 rats (23±6.7 pg/ml vs. 14±5.8 pg/ml, *p* = 0.022, 95% CI [1.5, 16]) (Fig. 5a). IL-5 levels were unaffected by *α*-Syn but were higher in DA.VRA4 compared to DA rats in the *α*-Syn group at 4 weeks (37±5.8 pg/ml vs. 25±11 pg/ml, *p* = 0.007, 95% CI [3.2, 22] (Fig. 5b).

DA.VRA4 rats with lower levels of *Ciita* had consistently higher levels of TNF in serum compared to DA in naïve (4.2±0.57 pg/ml vs. 3.3±0.56 pg/ml, *p* = 0.022, 95% CI [0.15, 1.6]), control- (4-week: 2.7±0.37 pg/ml vs. 2.0±0.16 pg/ml, *p* = 0.0012, 95% CI [0.31, 0.98] and 8-week: 4.3±0.58 pg/ml vs. 3.1±0.36 pg/ml, *p* = 0.00060, 95% CI [0.64, 1.8]) and *α*-Syn groups (4-week: 2.4±0.56 pg/ml vs. 1.9±0.22 pg/ml, *p* = 0.039, 95% CI [0.030, 1.0] and 8-week: 4.3±0.40 pg/ml vs. 3.1±0.43 pg/ml, *p* = 0.00020, 95% CI [0.71, 1.7]) (Fig. 5c).

## DISCUSSION

Studies investigating human cohorts and experimental models support a role for antigen presentation and adaptive immune responses in PD etiology [7, 11, 13–15]. However, there are contradictory findings on how resident and peripheral immune responses could contribute to or protect against neuropathology in *in vivo* PD models [13–15, 28–30]. Contributing factors to these discrepancies likely include difficulties in determining causality versus consequence in an ongoing pathological process, as well as the multiple different murine models used to study PD-related changes in the immune system. The use of transgenic models to model complex immune responses in human disease can be questioned. Therefore, we used a congenic rat model with naturally occurring *Ciita* alleles mediating differential expression of *Ciita* and MHC-genes in both rats and humans [16]. The CIITA protein regulates MHCII expression, and is a crucial link between antigen-presenting cells in the innate immune system and lymphocytes in the adaptive immune system. In a recent study, we showed that lower *Ciita* levels in rats are associated with increased susceptibility to *α*-Syn pathology and dopaminergic neurodegeneration [14, 15]. This strongly supports CIITA, MHCII and the process of antigen presentation to have causal impact on PD risk and outcome. The relative contribution of resident (brain) and peripheral (systemic) immune cells and cytokines in this process is, however, not known. Therefore, we characterized the effects of *Ciita* levels on local and peripheral immune populations and cytokines in the rAAV-*α*-Syn+PFF rat PD model. We used the congenic DA.VRA4 strain with variants in the *Ciita* gene mediating lower expression of MHCII and the background strain, DA. These congenic rats provide a physiologically highly relevant model to study the effects of antigen presentation on immune populations and PD-like pathology, especially since genetic variants in the human orthologue *CIITA* also regulate MHCII expression and are associated with susceptibility to rheumatoid arthritis, multiple sclerosis and myocardial infarction [16].

Results from different PD models confirm an influence of MHCII on PD-like pathology, but are contradictory in terms of the direction of effect from altered *Ciita* and MHCII expression. This could be explained by the heterogeneity in study design, including the use of different species (rats [14, 15, 29] or mice [13, 28, 30]), different models of PD/synucleinopathies (transgenic [30], rAAV-*α*-Syn [13, 14, 28, 29], or rAAV-*α*-Syn+PFF [15]) and different immunological models (knock-out (KO) models [13, 28, 30], nude rats [29], silencing through shRNA [13], or congenic strains [14, 15]). In response to *α*-Syn overexpression, *Mhc2*-KO, *Ciita*-KO and *Ciita* silencing protected mice against dopaminergic cell loss [13, 28] and led to reduction of T lymphocyte- and monocyte infiltration [13], microglial activation [28] and amount of MHCII+ microglia (CD45^dim^CD11b+) [13]. In contrast, *Mhc2*-KO in mice expressing human *α*-Syn with the A53T mutation (M83+/0) resulted in accelerated pathology in the brain and an overall reduction of T lymphocytes in the CNS after injection of PFF into the hindlimb [30]. Our data presented here and previously [14, 15] also indicate lower *Ciita* levels to increase susceptibility to PD-like pathology. However, the nature of the respective *in vivo* models is important to consider when interpreting these results. In humans, genetic mutations of *CIITA* or other crucial transcription factors for MHCII causes severe immunodeficiency (bare lymphocyte syndrome, BLS) [31]. It is therefore likely that *Ciita*- and *Mhc2*-KO models have compromised immune systems that limit their physiological relevance. Silencing of *Ciita* circumvents this issue, and might be the best model to study effects of immunotherapies, while our congenic model comes closest to the situation in humans where common *CIITA* variants regulate MHCII expression and disease susceptibility throughout life, including myeloid- and lymphocyte development and thymic selection. Of note, previous results on genetic association between *CIITA* and risk for multiple sclerosis, rheumatoid arthritis and myocardial infarction show a similar direction of effect as found in our congenic rat model, with the risk allele in *CIITA* being associated with lower expression of *CIITA*, *CD74*, *HLA-DRA* and *HLA-DQA1*. The combined genetic- and disease model employed in the current study has high construct validity (common *CIITA*/*Ciita* genetic variants regulate MHCII levels both in rats and humans) and high face validity (the rAAV-*α*-Syn+PFF rat model displays seeded *α*-Syn pathology, dopaminergic neurodegeneration, motor impairment, and neuroinflammation). Together, these characteristics allow for a good predictive validity of the model.

Using flow cytometry, we show that differential expression of *Ciita* affects the baseline levels of microglia and microglial MHCII-expression in naïve rats. Additionally, this study confirms previous semi-quantitative findings from brain immunostaining and RT-qPCR regarding microglial MHCII expression in response to *α*-Syn; lower *Ciita* levels in DA.VRA4 rats are associated lower levels of MHCII per cell [14, 15]. We hypothesize that increased numbers of MHCII+ microglia in naïve rats and region-specific differences in response to *α*-Syn (more MHCII+ microglia in striatum [15]) affect susceptibility and accelerate dopaminergic neurodegeneration through pathological spread of *α*-Syn, as we have previously reported an increased aggregation and propagation of *α*-Syn in DA.VRA4 rats, along with pathological *α*-Syn (pS129) co-localized within MHCII+ microglial cells in the rAAV-*α*-Syn+PFF model [15]. In contrast to our hypothesized model a recent study suggests that the main contributor to *α*-Syn-induced neurodegeneration is border-associated macrophages (BAMs) rather than microglia [32]. In the current study we were unable to investigate any specific contribution of infiltrating BAMs in the brain parenchyma since they are indistinguishable from microglia based on CD45- and CD11b-expression [33].

Compared to studies using *α*-Syn nigral overexpression or striatal PFF injection in mice [13, 21, 34], we found very limited numbers of brain-infiltrating macrophages/monocytes and lymphocytes in the rAAV-*α*-Syn+PFF rat model. Among live cells analyzed from brain tissue, 93–97% were CD45^dim^CD11b+ microglia and only 0.5–1.5% CD45^high^CD11b+ macrophages/monocytes. However, a large majority (70–85%) of macrophages/monocytes but a minority (5–15%) of the microglia were MHCII+, indicating that infiltrating macrophages/monocytes might still play an important role in CNS antigen presentation. The low number of infiltrating macrophages/monocytes and lack of differences between *α*-Syn and control groups in this model are contradictory to a previous study in a nigral *α*-Syn overexpression model in mice, where PD-like pathology was mainly driven by infiltrating monocytes [35]. In addition, *Ciita* levels did not affect the number of infiltrating macrophages/monocytes or lymphocytes in our model, while KO and silencing of *Ciita* have been reported to greatly reduce both monocyte and lymphocyte infiltration in mice overexpressing *α*-Syn in SN [13]. Additionally, we show that naïve DA.VRA4 rats with lower *Ciita* levels than DA also have a higher percentage of MHCII+ circulating cells of the myeloid lineage and that these circulating myeloid cells have lower MHCII expression compared to DA in the *α*-Syn group at 4 weeks. The MHCII expression on circulating myeloid cells were also highest at *α*-Syn 8 weeks in both DA.VRA4 and DA.

We found few infiltrating T lymphocytes (CD45+CD3+) in brain tissue, and the amounts were independent of *Ciita* levels. In blood, DA.VRA4 rats with lower *Ciita* levels had fewer T lymphocytes compared to DA at 4 weeks after rAAV-*α*-Syn+PFF. There was also a lower CD4/CD8 ratio in naïve DA.VRA4 rats compared to DA. Studies in other *α*-Syn-based PD models indicate both detrimental and protective roles of lymphocytes. Neurodegeneration-promoting effects are supported by findings that mice lacking lymphocytes (*Rag1* KO) were protected against dopaminergic cell loss in SN, that lymphocyte reconstitution resulted in dopaminergic cell loss comparable to wild type mice [36] and that CD4 KO protected against neurodegeneration in the SN and inhibited myeloid activation [37]. In contrast, protective effects of lymphocytes have been reported in a striatal PFF model, where adoptive transfer of CD4+ lymphocytes to immunocompromised mice reduced *α*-Syn pathology [38]. More detailed studies are required to delineate if the observed differences in the current study are of biological relevance to susceptibility and/or progression of PD-like pathology.

Although recent genetic studies point at MHCII, but not MHCI, genes associated to PD [7], neuronal MHCI expression [39], CD8+ T lymphocyte infiltration [40] and reactivity to *α*-Syn [11] could potentially play a role in PD susceptibility. Studies on lymphocyte populations in PD patients are, however, inconclusive. Different studies have reported no difference [41] or decrease [42] in CD4+ and CD8+ populations, a reduction in effector and regulatory T lymphocytes [41], a lower CD4/CD8 ratio [43] and an overall decrease in circulating CD4+ lymphocyte subpopulations due to decreased levels of T-helper (Th) 2, Th17 and regulatory lymphocytes [44]. Other research suggests an overall decrease in circulating lymphocytes with increased Th1 and Th17 but decreased Th2 and regulatory T lymphocytes [45] or no changes in Th1 and Th2 subsets but an increase in the Th17 lymphocyte population [46]. In addition to differences in population sizes and ratios, functional studies indicate alterations in lymphocyte populations in PD, including deficits in migratory capacity of CD4+ T lymphocytes from PD patients [47] and impaired suppressor functions of T regulatory cells in PD, which could be restored by ex vivo expansion [41]. A third study reported that PD disease severity was associated with higher activation levels of T lymphocytes in response to phytohemagglutinin stimulation [42]. In light of these findings from PD patients, a limitation of our study is not assessing *Ciita* effects on MHCI expression, another is the low number of lymphocytes detected in brain tissue that limited analyses of sub-populations of infiltrating T lymphocytes.

In addition to altered immune cell profiles, we analyzed CSF and serum levels of cytokines. Although few differences were found, there are several links to PD for the cytokines that differed depending on strain and/or rAAV-*α*-Syn+PFF injection. Elevated IL-6 levels in CSF have been observed in PD patients [48] and we found that rAAV-*α*-Syn+PFF injection resulted in increased CSF IL-6 levels in both DA and DA.VRA4 rats. IL-10 has previously been shown to be neuroprotective and reduce microglial activation in toxin models of PD [49]. In contrast, AAV mediated overexpression of viral IL-10 (IL-10 lacking immunostimulatory function [50]) in the spinal cord of M83+/0 mice subjected to intramuscular injection of PFF resulted in a reduced life span and exacerbated microglial activation, *α*-Syn pathology and spinal cord cell death [51]. We found higher IL-10 levels in CSF from DA.VRA4 rats compared to DA in response to *α*-Syn at 8-weeks. To assess if the contribution of IL-10 in our disease model is protective or contributes to PD-like progression requires further investigation. IL-1β levels in blood have been reported in multiple studies to be increased in PD patients [52] and to correlate with disease progression [53]. IL-1β levels have also been shown to influence the NLRP3 inflammasome which contributes to neurodegeneration in a 6-OHDA mouse model of PD [54]. We found higher levels of IL-1β in naïve DA.VRA4 vs. DA and a significant upregulation in DA.VRA4 rats in response to *α*-Syn. It is possible that elevated IL-1β levels exacerbates the susceptibility and progression of PD-like pathology in the DA.VRA4 rats. Blocking IL-1β signaling by IL-1 receptor antagonist has been shown to be neuroprotective in a PD model using sub-toxic doses of LPS injected into the SN combined with striatal 6-OHDA lesion [55]. IL-5 has been reported to be elevated in CD4+ Th2 lymphocytes from PD patients stimulated with *α*-Syn peptides ex vivo [11]. We found higher IL-5 levels in serum from DA.VRA4 compared to DA in the *α*-Syn group at 4 weeks but determining the source of IL-5 requires e.g., functional ex vivo studies. However, the elevated IL-5 levels at 4 but not 8 weeks corresponds to the human situation were *α*-Syn specific T lymphocyte reactivity is mainly restricted to subclinical and early PD [12].

Interestingly, we found consistently higher TNF levels in serum but not CSF in DA.VRA4 compared to DA rats. Other studies have shown that intrathecal neutralization of soluble TNF by dominant-negative protein (DN-TNF) in SN, but not striatum, protects from 6-OHDA- and LPS-induced degeneration of rat nigral dopaminergic neurons and neuroinflammation *in vivo* and *in vitro* [56, 57]. The neuroprotective effect of viral vector-mediated expression of DN-TNF was still present when administered 2 weeks after 6-OHDA lesion [58], while peripheral administration of DN-TNF was shown to cross the blood-brain barrier, reduce astrocyte- and microglial number in SN and to reduce neurodegeneration when administered 3 days, but not 14 days, after striatal 6-OHDA lesion [59]. Based on this data, TNF could exert direct and indirect effects on neurons, astrocytes, microglia and T lymphocytes. Further, it is possible that higher levels of TNF in DA.VRA4 rats affect the susceptibility to PD-like pathology and together with IL-1β and IL-5 exacerbates *α*-Syn pathological spread and dopaminergic neurodegeneration. Further investigation is necessary to provide a mechanistic view, i.e. by assessing the effects of systemic TNF inhibition on neuroinflammation, neurodegeneration and *α*-Syn pathology in the rAAV-*α*-syn+PFF model in the DA.VRA4 rats with elevated TNF serum levels.

As all models, the rAAV-*α*-Syn+PFF PD rat model has both strengths and limitations. Similar to other models using intracranial injections, the physical damage and blood-brain barrier disruption could cause changes in immune populations, independent of what is injected. Advantages of the combined rAAV-*α*-Syn+PFF model compared to PFF or rAAV-*α*-Syn alone include a faster PD-like disease progression and an *α*-Syn pathology that is proteinase K resistant, much like Lewy bodies in PD brains [15, 17, 60, 61]. In order to control for immune responses not related to *α*-Syn, we injected the same rAAV vector but without the human *α*-Syn transgene in SN and vehicle in striatum for the control groups. We chose a control vector without a transgene since we and others have observed that the commonly used rAAV-GFP control vector elicits a neuroinflammatory response [14, 15, 35] and dopaminergic neurodegeneration [15, 60]. As control for PFF, we chose to use vehicle, since we have found that bovine serum albumin elicits a neuroinflammatory response [15] and other studies report that *α*-Syn monomers and saline are comparable controls for the PFF model in rats [20]. This design, however, means that we cannot determine to what extent the expression of a foreign protein or possible contamination with endotoxin in the PFF preparation affect the results. Another aspect to consider is the timepoints selected. We have previously investigated the effects of differential *Ciita* expression on PD-like *α*-Syn pathology, neurodegeneration and neuroinflammation at 8-weeks in the rAAV-*α*-Syn+PFF model [15] and at 12-weeks in the rAAV-*α*-Syn model [14]. However, studies have shown that there is an inflammatory response ongoing prior to neurodegeneration in animal models [19–21] and in PD patients [11]. We chose to include two timepoints (4- and 8 weeks post nigral injection), but acknowledge that the results might differ at other timepoints. A technical limitation is that we used entire hemispheres for analyses of brain tissue by flow cytometry. Although *α*-Syn pathology and MHCII+ microglial cells are widespread in the brain in the rAAV-*α*-Syn+PFF model [15], it is possible that region-specific differences affected by *Ciita* levels or responses to *α*-Syn are larger than what was reflected in entire hemispheres.

Even though there is substantial evidence of the involvement of antigen presentation in PD based on genetic association studies and elevated MHCII levels at the site of neurodegeneration [3–5, 7, 62, 63], sufficient knowledge on the role of MHCII in disease etiology is lacking. Association between *HLA* alleles and PD risk has been found for expression quantitative trait loci (eQTL) [4–6], non-coding variants [7, 64] and non-synonymous coding variants [7]. The heterogeneity and strong linkage within the *HLA* make discrimination between these effects difficult. In light of previous findings and the current study that point towards *Ciita*-mediated effects on PD-like pathology, we hypothesize that alleles affecting *Ciita* expression interact with non-coding risk-*HLA* alleles affecting MHCII expression (eQTLs). Further, *Ciita* expression could modify the effect of coding risk-*HLA* alleles by affecting their expression levels. In addition, *HLA* alleles have been reported to interact with other non-genetic factors, including pyrethroids and smoking [5, 65] and *Ciita* could potentially be part of such interactive effects.

In conclusion, despite significant differences in PD-relevant neurodegenerative and behavioral phenotypes, we observed only subtle differences in immune cell populations and cytokine profiles between the more susceptible DA.VRA4 and the more resistant DA rats, the most consistent being higher levels of serum TNF in DA.VRA4. To determine if and how these immune effects are causally related to the increased susceptibility to *α*-Syn-induced PD-like pathology, further studies are required, e.g., with specific cytokine inhibitors. Our work together with other experimental and human studies highlight the complexity and importance of understanding the link between innate and adaptive immune responses in PD. Our work also suggests that future immunomodulatory therapies for PD could be efficient without major impact on the immune system as a whole.

## Supplementary Material

Supplementary Material

## Data Availability

The data supporting the findings of this study are available on request from the corresponding author.
