# Supplementary material for: Ciita Regulates Local and Systemic Immune Responses in a Combined rAAV-α-synuclein and Preformed Fibril-Induced Rat Model for Parkinson’s Disease
Source: J Parkinsons Dis. 2024 Jun 4;14(4):693–711. doi: 10.3233/JPD-240062 (PMC11191526; doi:10.3233/JPD-240062)
Supplement: Supplementary Material [file jpd-14-jpd240062-s001.pdf]

Supplementary Material

***Ciita* Regulates Local and Systemic Immune Responses in a Combined rAAV-α-synuclein and Preformed Fibril-Induced Rat Model for Parkinson's Disease**

Supplementary Table 1. Cytokine levels in CSF from naïve DA and DA.VRA4 rats. Lower limit of quantification (LLOQ) is specified in brackets after each cytokine. Data presented as mean±SD. ND=non-detected. Unpaired Student's t-test.

CSF cytokines (pg/ml)	DA Naïve (n=5)	DA.VRA4 Naïve (n=6)
**IFNγ (39.7)**	4.59±0.887	4.96±1.17
**IL-10 (163)**	7.02±1.67	7.70±1.04
**IL-13 (12.5)**	ND	ND
**IL-1β (102)**	16.1±6.22	15.5±3.26
**IL-4 (8.00)**	1.10±0.606	1.23±0.577
**IL-5 (82.0)**	37.6±12.3	36.5±11.5
**IL-6 (96.9)**	87.6±24.4	86.3±24.6
**KC/GRO (21.7)**	72.7±14.4	87.8±8.69
**TNF (9.10)**	1.48±0.337	1.57±0.306

Supplementary Table 2. No differences in cytokine levels in CSF from DA and DA.VRA4 rats in the α-Syn group at 4 weeks. Lower limit of quantification (LLOQ) is specified in brackets after each cytokine. Data presented as mean±SD. α-Syn=rAAV-α-Syn+PFF. Control=rAAV-(-)+DPBS. ND, non-detected. Two-way ANOVA with Šídák multiple comparison test was used to compare strains (DA vs. DA.VRA4) and α-syn model (a-Syn vs. control).

**CSF cytokines at 4 weeks (pg/ml)**	**DA Control (n=7)**	**DA.VRA4 Control (n=7)**	**DA α-Syn (n=7)**	**DA.VRA4 α-Syn (n=8)**
**IFNγ (39.7)**	2.63±1.25	3.50±1.89	2.75±1.04	3.44±0.924
**IL-10 (163)**	4.67±1.74	3.71±1.23	5.65±2.40	5.03±2.29
**IL-13 (12.5)**	ND	ND	ND	ND
**IL-1β (102)**	ND	ND	ND	9.69±9.95
**IL-4 (8.00)**	0.415±0.268	ND	ND	ND
**IL-5 (82.0)**	23.1±4.88	21.8±4.30	23.4±9.15	24.0±8.82
**IL-6 (96.9)**	51.9±12.0	42.4±16.9	55.1±25.1	55.0±40.0
**KC/GRO (21.7)**	75.9±19.2	92.7±21.3	78.5±45.1	108±43.3
**TNF (9.10)**	0.954±0.326	1.21±0.338	1.13±0.550	2.21±2.24

Supplementary Table 3. Cytokine levels in CSF from DA and DA.VRA4 rats in α-Syn or control groups at 8 weeks. Lower limit of quantification (LLOQ) is specified in brackets after each cytokine. Data presented as mean±SD. α-Syn, rAAV-α-Syn+PFF. Control, rAAV-(-)+DPBS. ND, non-detected. Two-way ANOVA with Šídák multiple comparison test was used to compare strains (DA vs. DA.VRA4) and α-syn model (a-Syn vs. control). $^{**}p < 0.01$ (DA vs. DA.VRA4). $^{\#\#}p < 0.01$ and $^{\#\#\#}p < 0.001$ (a-Syn vs. control).

**CSF cytokines at 8 weeks (pg/ml)**	**DA Control (n=7)**	**DA.VRA4 Control (n=8)**	**DA α-Syn (n=8)**	**DA.VRA4 α-Syn (n=8)**
**IFNγ (39.7)**	ND	ND	1.10±0.597	1.52±2.34
**IL-10 (163)**	ND	ND	1.75±0.738	3.16±1.07 **
**IL-13 (12.5)**	ND	ND	ND	ND
**IL-1β (102)**	ND	ND	ND	ND
**IL-4 (8.00)**	ND	ND	0.477±0.331	0.683±0.334
**IL-5 (82.0)**	9.60±4.75	ND	12.7±2.76	12.4±5.40
**IL-6 (96.9)**	12.0±12.0	6.56±7.65	44.6±20.8 $^{\#\#\#}$	31.0±11.2 $^{\#\#}$
**KC/GRO (21.7)**	50.0±25.1	79.9±67.1	60.1±30.1	51.8±6.37
**TNF (9.10)**	0.198±0.190	0.267±0.199	0.407±0.183	0.431±0.276

Supplementary Table 4. Cytokine levels in serum from naïve DA and DA.VRA4 rats. Lower limit of quantification (LLOQ) is specified in brackets after each cytokine. Data presented as mean±SD. ND=non-detected. Unpaired Student's t-test. * $p < 0.05$.

**Serum cytokines (pg/ml)**	**DA Naïve (n=6)**	**DA.VRA4 Naïve (n=6)**
**IFNγ (39.7)**	ND	ND
**IL-10 (163)**	11.6±3.09	13.2±6.14
**IL-13 (12.5)**	1.10±1.09	1.15±0.695
**IL-1β (102)**	10.8±8.35	28.5±14.2 *
**IL-4 (8.00)**	0.449±0.287	ND
**IL-5 (82.0)**	17.9±6.04	24.4±12.1
**IL-6 (96.9)**	ND	11.1±6.13
**KC/GRO (21.7)**	289±203	216±108
**TNF (9.10)**	3.34±0.564	4.22±0.567 *

Supplementary Table 5. Cytokine levels in serum from DA and DA.VRA4 rats in α-Syn and control groups at 4 weeks. Lower limit of quantification (LLOQ) is specified in brackets after each cytokine. Data presented as mean±SD. α-Syn, rAAV-α-Syn+PFF. Control, rAAV-(-)+DPBS. ND, non-detected. Two-way ANOVA with Šídák multiple comparison test was used to compare strains (DA vs. DA.VRA4) and α-syn model (a-Syn vs. control). * $p < 0.05$ and ** $p < 0.01$ (DA vs. DA.VRA4). # $p < 0.05$ (a-Syn vs. control).

**Serum cytokines at 4 weeks (pg/ml)**	**DA Control (n=7)**	**DA.VRA4 Control (n=7)**	**DA α-Syn (n=7)**	**DA.VRA4 α-Syn (n=7)**
**IFNγ (39.7)**	ND	ND	ND	ND
**IL-10 (163)**	14.8±4.07	10.6±3.37	15.9±4.98	13.8±3.77
**IL-13 (12.5)**	ND	ND	ND	1.13±1.07
**IL-1β (102)**	ND	14.4±5.81	ND	23.2±6.70 #
**IL-4 (8.00)**	0.524±0.160	0.438±0.182	0.538±0.346	0.465±0.208
**IL-5 (82.0)**	25.1±6.85	28.7±4.18 **	24.8±10.8	37.3±5.78
**IL-6 (96.9)**	18.8±18.1	ND	20.4±10.4	20.9±16.2
**KC/GRO (21.7)**	334±312	240±69.3	163±48.2	221±62.4
**TNF (9.10)**	2.03±0.162	2.68±0.373 **	1.89±0.217	2.42±0.561 *

Supplementary Table 6. Cytokine levels in serum from DA and DA.VRA4 rats in α-Syn and control groups at 8 weeks. Lower limit of quantification (LLOQ) is specified in brackets after each cytokine. Data presented as mean±SD. α-Syn, rAAV-α-Syn+PFF. Control, rAAV-(-)+DPBS. ND, non-detected. Two-way ANOVA with Šídák multiple comparison test was used to compare strains (DA vs. DA.VRA4) and α-syn model (a-Syn vs. control). ***p < 0.001 (DA vs. DA.VRA4).

**Serum cytokines at 8 weeks (pg/ml)**	**DA Control (n=7)**	**DA.VRA4 Control (n=7)**	**DA α-Syn (n=7)**	**DA.VRA4 α-Syn (n=7)**
**IFNγ (39.7)**	7.33±12.0	ND	15.7±21.5	11.9±24.4
**IL-10 (163)**	ND	ND	ND	ND
**IL-13 (12.5)**	1.36±0.980	1.31±1.17	1.48±0.837	1.70±0.820
**IL-1β (102)**	36.8±30.2	44.2±19.5	24.1±16.0	47.0±27.8
**IL-4 (8.00)**	ND	ND	ND	ND
**IL-5 (82.0)**	26.7±19.0	38.1±31.6	24.8±12.1	35.2±20.0
**IL-6 (96.9)**	43.7±38.2	45.6±31.4	62.6±34.0	73.7±31.6
**KC/GRO (21.7)**	257±135	305±99.6	215±114	283±96.2
**TNF (9.10)**	3.12±0.362	4.33±0.584 ***	3.10±0.432	4.30±0.393 ***

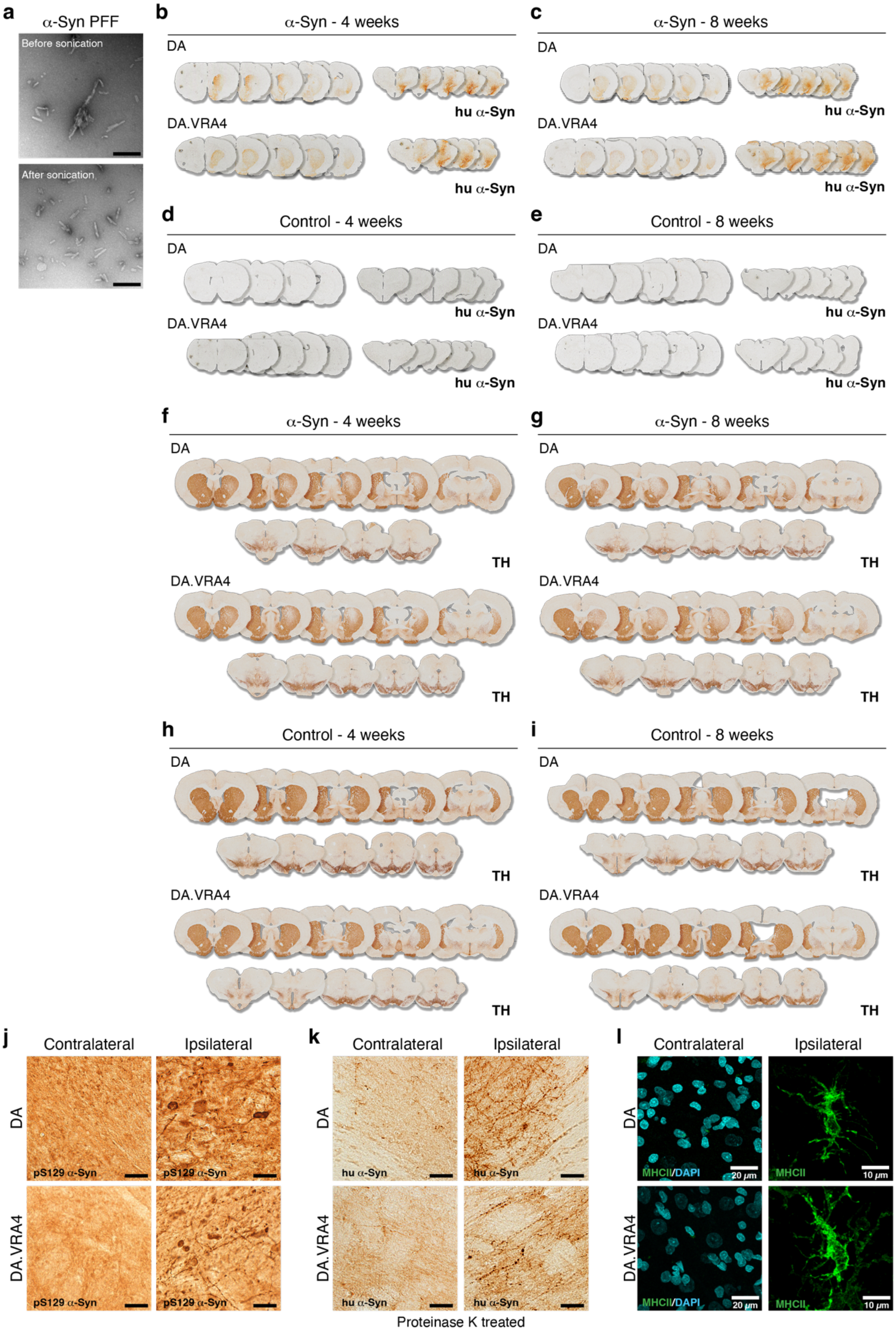

a
α-Syn PFF
Before sonication
After sonication
b
α-Syn - 4 weeks
DA
hu α-Syn
DA.VRA4
hu α-Syn
c
α-Syn - 8 weeks
DA
hu α-Syn
DA.VRA4
hu α-Syn
d
Control - 4 weeks
DA
hu α-Syn
DA.VRA4
hu α-Syn
e
Control - 8 weeks
DA
hu α-Syn
DA.VRA4
hu α-Syn
f
α-Syn - 4 weeks
DA
TH
DA.VRA4
TH
g
α-Syn - 8 weeks
DA
TH
DA.VRA4
TH
h
Control - 4 weeks
DA
TH
DA.VRA4
TH
i
Control - 8 weeks
DA
TH
DA.VRA4
TH
j
Contralateral
Ipsilateral
DA
DA.VRA4
pS129 α-Syn
k
Contralateral
Ipsilateral
DA
DA.VRA4
hu α-Syn
Proteinase K treated
l
Contralateral
Ipsilateral
DA
DA.VRA4
MHCII/DAPI
MHCII
20 μm
10 μm

Supplementary Figure 1. Representative images of human a-Syn and TH immunostaining in the brain of a-Syn and control groups. a. TEM images of a-Syn PFF before (top) and after (bottom) sonication; sonicated PFF were used for striatal seeding. Scale bar = 200 nm. Expression of human a-Syn was detected at 4- (b) and 8-weeks (c) in the a-Syn groups. d-e. Control groups did not show any signal for human a-Syn at 4- or 8-weeks. d. Loss of TH signal was evident at 4- (f) and 8-weeks (g) in the a-Syn groups in both DA and DA.VRA4 rats. No TH loss was observed in control groups at 4- (h) or 8-weeks (i). Unilateral injection of rAAV-a-Syn+PFF resulted in pathological forms of a-Syn determined by pS129 a-Syn (j) and proteinase K resistant human a-Syn (k) stainings. l. Upregulation of MHCII on microglia was observed in the ipsilateral midbrain. Representative images in the a-Syn group at 8 weeks. j-k. Scale bar = 20 µm. l. Contralateral scale bar = 20 µm, ipsilateral scale bar = 10 µm. α-Syn=rAAV-α-Syn+PFF. Control=rAAV-(-)+DPBS.

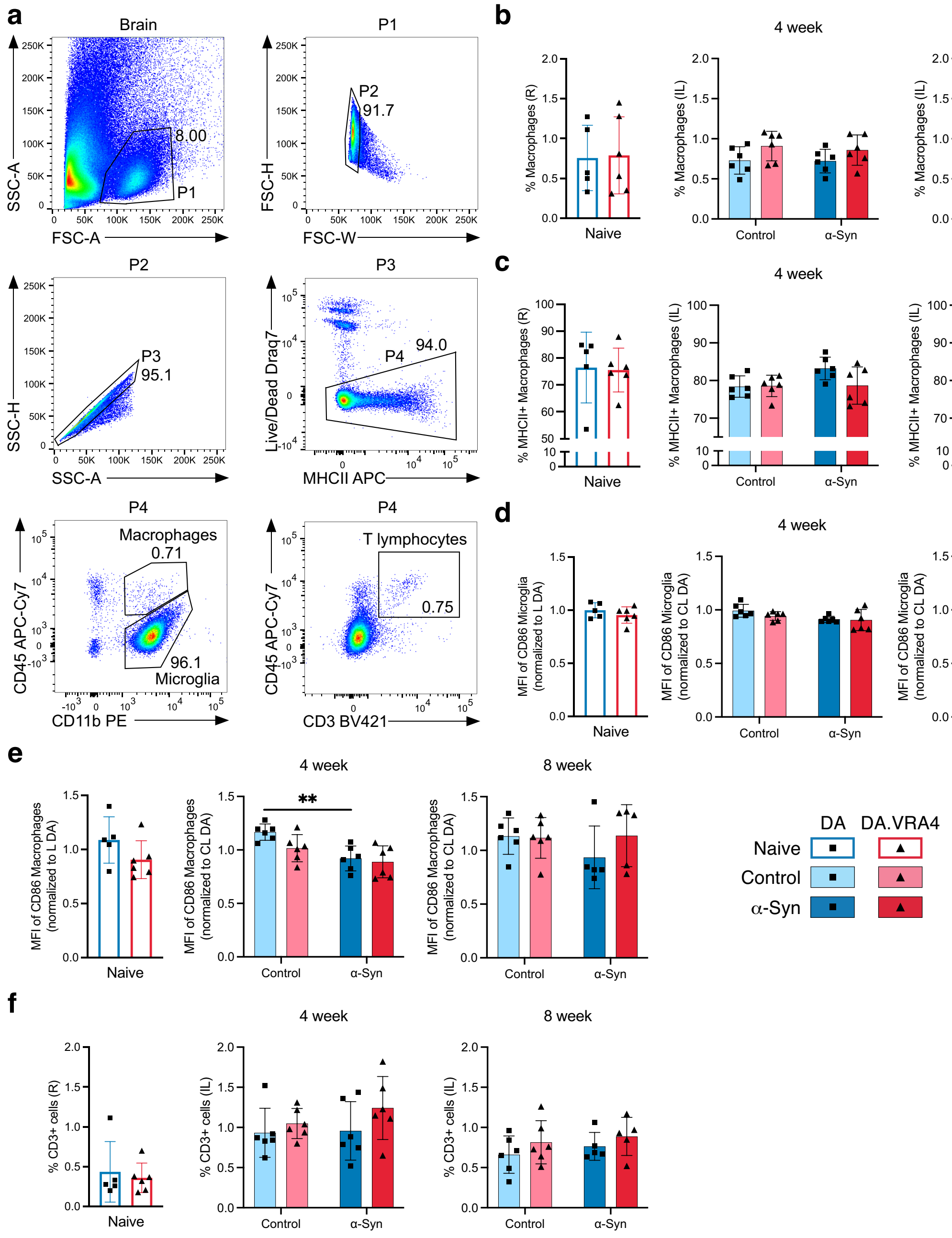

Supplementary Figure 2. rAAV-a-Syn+PFF injection does not change brain-infiltrating macrophage/monocyte number, MHCII+ macrophage numbers or infiltration of lymphocytes. a. Complete gating strategy of brain hemispheres for flow cytometry. b. Quantification of macrophages ($CD45^{high}CD11b+$) in right (R)/ipsilateral (IL) hemispheres. c. Quantification of MHCII+ microglia in the R/IL hemispheres. d. Quantification of relative median fluorescence intensity (MFI) of CD86 on microglia in R/IL hemispheres. At each recording session, R/IL MFI-values were normalized to the mean MFI-values in left(L)/contralateral (CL) hemispheres from DA rats. e. MFI of CD86 on macrophages in R/IL hemispheres. f. CD3+ cells in IL hemisphere do not change in response to rAAV-α-Syn+PFF. Naïve (DA n=5, DA.VRA4

n=6), 4-week; control (DA n=6, DA.VRA4 n=6) and a-Syn (DA n=6, DA.VRA4 n=6), 8-week; control (DA n=6, DA.VRA4 n=6) and a-Syn (DA n=5, DA.VRA4 n=5). Data presented as mean ± SD with individual values. α-Syn=rAAV-α-Syn+PFF. Control=rAAV-(-)+DPBS. Naïve DA and DA.VRA4 rats were compared by unpaired Student's t-test. Two-way ANOVA with Šídák multiple comparison test was used to compare strains (DA vs. DA.VRA4) and experimental groups (a-Syn vs. control) at 4- and 8-weeks. **$p < 0.01$

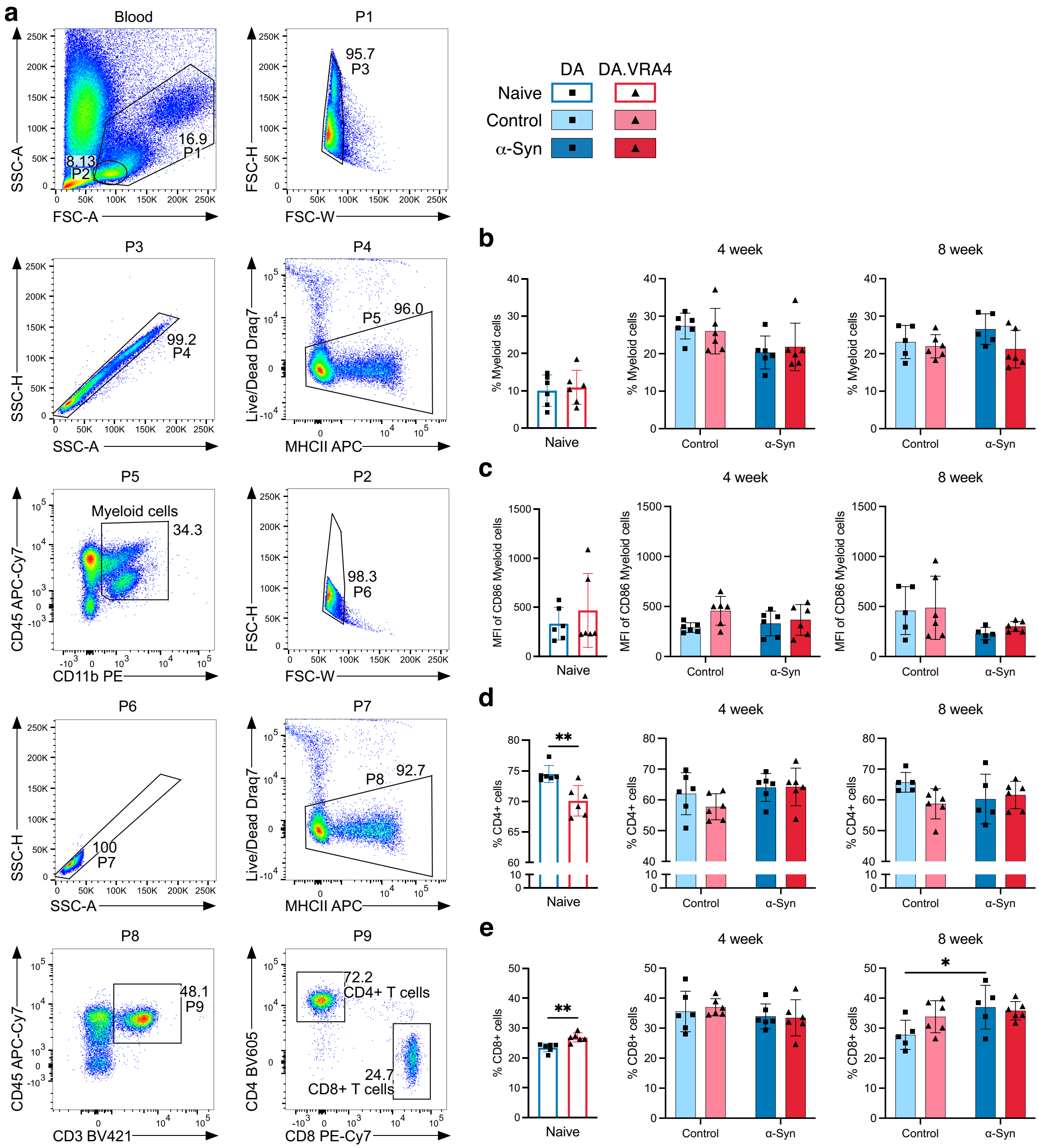

Supplementary Figure 3. Circulating myeloid numbers and myeloid CD86 expression does not change in response to rAAV-a-Syn+PFF. a. Gating strategy for blood flow cytometry. b. Percentage of myeloid cells (CD45+CD11b+) in blood. c. MFI of CD86 on myeloid cells. d. Percentage of CD4+ T lymphocytes. e. Percentage of CD8+ T lymphocytes. b-e. Naïve (DA n=6, DA.VRA4 n=6), 4 week; control (DA n=6, DA.VRA4 n=6) and a-Syn (DA n=6, DA.VRA4 n=6), 8 week; control (DA n=5, DA.VRA4 n=6) and a-Syn (DA n=5, DA.VRA4 n=6). Data presented as mean ± SD with individual values. α-Syn=rAAV-α-Syn+PFF. Control=rAAV-(-)+DPBS. Naïve DA and DA.VRA4 rats were compared by unpaired Student's t-test. Two-way ANOVA with Šídák multiple comparison test was used to compare strains (DA vs. DA.VRA4) and experimental groups (a-Syn vs. control) at 4- and 8-weeks. * $p < 0.05$ and ** $p < 0.01$.
